# Biodegradable, Efficient, and Breathable Multi‐Use Face Mask Filter

**DOI:** 10.1002/advs.202003155

**Published:** 2021-01-29

**Authors:** Sejin Choi, Hyeonyeol Jeon, Min Jang, Hyeri Kim, Giyoung Shin, Jun Mo Koo, Minkyung Lee, Hye Kyeong Sung, Youngho Eom, Ho‐Sung Yang, Jonggeon Jegal, Jeyoung Park, Dongyeop X. Oh, Sung Yeon Hwang

**Affiliations:** ^1^ Research Center for Bio‐Based Chemistry Korea Research Institute of Chemical Technology (KRICT) Ulsan 44429 Republic of Korea; ^2^ Department of Polymer Engineering Pukyong National University Busan 48513 Republic of Korea; ^3^ Advanced Materials and Chemical Engineering University of Science and Technology (UST) Daejeon 34113 Republic of Korea

**Keywords:** biodegradability, chitosan, face masks, particulate matter, polybutylene succinate

## Abstract

The demand for face masks is increasing exponentially due to the coronavirus pandemic and issues associated with airborne particulate matter (PM). However, both conventional electrostatic‐ and nanosieve‐based mask filters are single‐use and are not degradable or recyclable, which creates serious waste problems. In addition, the former loses function under humid conditions, while the latter operates with a significant air‐pressure drop and suffers from relatively fast pore blockage. Herein, a biodegradable, moisture‐resistant, highly breathable, and high‐performance fibrous mask filter is developed. Briefly, two biodegradable microfiber and nanofiber mats are integrated into a Janus membrane filter and then coated by cationically charged chitosan nanowhiskers. This filter is as efficient as the commercial N95 filter and removes 98.3% of 2.5 µm PM. The nanofiber physically sieves fine PM and the microfiber provides a low pressure differential of 59 Pa, which is comfortable for human breathing. In contrast to the dramatic performance decline of the commercial N95 filter when exposed to moisture, this filter exhibits negligible performance loss and is therefore multi‐usable because the permanent dipoles of the chitosan adsorb ultrafine PM (e.g., nitrogen and sulfur oxides). Importantly, this filter completely decomposes within 4 weeks in composting soil.

## Graphical abstract

An eco‐friendly face mask filter with high‐level functionality is developed. Made only of biodegradable materials, it completely decomposes in the soil, thereby providing a fundamental waste problem solution. Moreover, the filter is a practical alternative to conventional disposable filters by integrating the physical sieving role of a poly(butylene succinate) fiber mat and the permanent electrostatic adsorption roles of chitosan nanowhiskers.

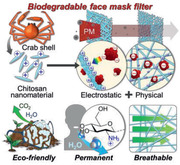

The current unprecedented coronavirus pandemic (COVID‐19) is driving a huge demand for face masks.^[^ ^1a^ ^]^ The World Health Organization (WHO) estimates that 89 million medical masks are required every month of this year.^[^ ^1b^ ^]^ Not only highly efficient N95 level masks are required for healthcare professionals, but general masks for all individuals have also become an essential daily equipment for protection against this respiratory contagion.^[^ ^1c^ ^]^ Moreover, concerned ministries have strongly recommended the daily use of single‐use disposable masks,^[^ ^1d^ ^]^ which has led to environmental issues associated with large volumes of mask waste.

As particulate matter (PM) is currently the most problematic air pollution issue, face masks have become the most effective countermeasure available to the individual. PM, which is classified as PM_2.5_ and PM_10_ based on particle size (2.5 and 10 µm, respectively), severely impacts the natural environment in various ways,^[^ ^2a–d^ ^]^ as well as the quality of human life.^[^ ^2e–g^ ^]^ Annually, PM is responsible for 4.2 million deaths and 103.1 million disability‐adjusted life‐years.^[^ ^2h^ ^]^ PM_2.5_ is an especially serious threat to health and is officially designated as a Group I carcinogen.^[^ ^2i^ ^]^ Therefore, it is timely and significant to research and develop an efficient face mask filter in terms of air permeability and PM removal.^[^ ^3a,b^ ^]^

In general, a conventional fibrous filter captures PM in two different ways: through nanofiber‐based physical sieving and microfiber‐based electrostatic adsorption (**Figure** **1**a). The use of a nanofiber‐based filter, especially an electrospun nanofiber mat, has been verified to be an effective strategy for PM removal, a consequence of broad material availability and controllable product structures.^[^ ^3a,c–e^ ^]^ The nanofiber mat is able to remove size‐targeted particles, which is attributed to the size difference between the particles and pores.^[^ ^3f,g^ ^]^ However, nanoscale fibers need to be densely stacked to form extremely small pores, which is detrimental to comfortable human breathing due to the associated high pressure differential. In addition, the small pores inevitably suffer from relatively fast blockage.

**Figure 1:**
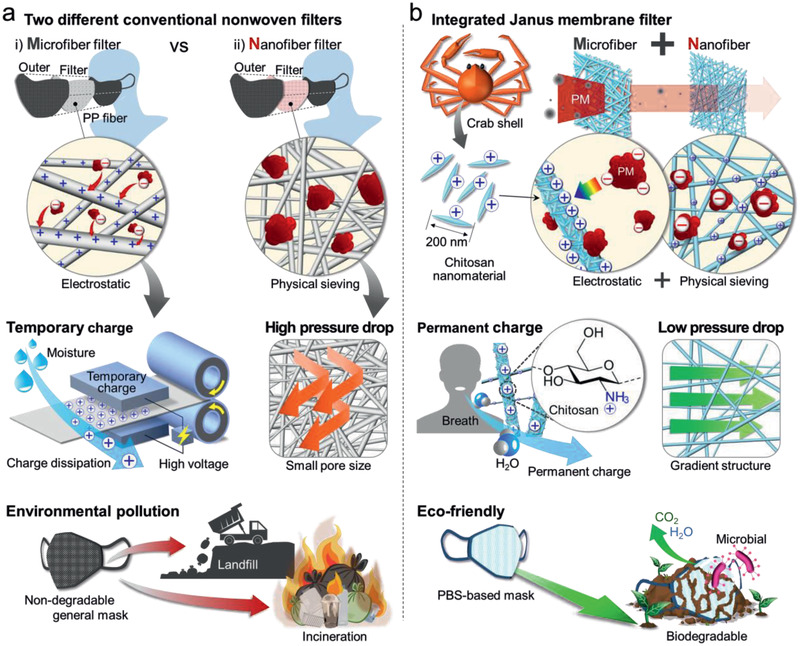
Filtration mechanisms for particulate matter (PM), characteristics in use, and environmental impact of conventional and developed mask filters. a) Two different representative PM capturing mechanisms of conventional nonwoven filters, and their consequential shortcomings: temporary charge of a microfiber‐based electrostatic filter, the high pressure drop of a nanofiber‐based physical filter, and environmental pollution (as the masks are disposable). b) Outstanding characteristics of the developed chitosan‐coated PBS nanofiber/microfiber integrated Janus membrane filter: permanently preserved ionic charges, low pressure drop facilitates comfortable breathing by the user, and biodegradability (Movie S1, Supporting Information).

On the other hand, a melt‐blown microfiber mat, which is electrostatically charged by a high‐energy electric field, captures extremely small particles through electrostatic adsorption.^[^ ^4a–d^ ^]^ The N95 mask, as a representative example, is a particulate‐filtering face‐piece respirator that meets the requirement of the U.S. National Institute for Occupational Safety and Health, in that it filters at least 95% of airborne particles. This type of filter adsorbs ultrafine PM that is usually composed of anionic substances, such as SO_4_^2−^ and NO_3_^−^, through strong electrostatic attraction. However, the surface electrostatic charge of the fiber mat is readily dissipated in a humid environment, such that found in moist human breath,^[^ ^4e,f^ ^]^ leading to a decline in adsorption ability.

To further enhance filtration performance or tackle the trade‐off between removal efficiency and pressure drop, nanofiber‐ and microfiber‐based filters are incorporated with high‐*k* materials, such as carbon materials, metal‐organic frameworks, and polytetrafluoroethylene nanoparticles.^[^ ^4g–i^ ^]^ However, the uncertain biological toxicity of these additives and charge dissipation remain inevitable issues.^[^ ^4j^ ^]^ In particular, both types of conventional filter are commonly non‐degradable and, as a consequence, are eventually buried in landfills or incinerated after use. Hence, the development of an improved face mask filter that addresses these waste problems and simultaneously captures PM in a satisfactory and highly functional manner is a significant current demand.

To address the abovementioned issues, we fabricated a Janus membrane filter integrated with poly(butylene succinate)‐based (PBS‐based)^[^ ^5a^ ^]^ microfiber and nanofiber mats. The Janus membrane filter was coated with chitosan nanowhiskers (CsWs)^[^ ^5b^ ^]^ (Figure 1b). PBS, which is known to be a representative biodegradable polymer, was electrospun to produce microfiber and nanofiber nonwovens. The nanoscale fibers physically capture PM, while the microscale nanofibers reduce the pressure drop and act as the CsW framework. Chitosan, a bio‐based material, has been shown to have favorable biological properties, including biocompatibility, biodegradability, and relatively low toxicity,^[^ ^5c,d,e^ ^]^ which reduces anxiety associated with accidental inhalation by the user.^[^ ^5f^ ^]^ Moreover, chitosan possesses cationic sites and polar amide groups,^[^ ^5g,h^ ^]^ which attract polar ultrafine PM (e.g., SO_4_^2−^ and NO_3_^−^) even under humid conditions.

Herein, we report a biodegradable, highly efficient, moisture‐resistant, and low pressure drop face mask filter based on easy‐to‐obtain biodegradable materials. The CsW‐coated microfiber/nanofiber‐integrated filter presented a high PM_2.5_ removal efficiency (up to 98%) due to a combination of physical sieving and electrostatic adsorption, while delivering a maximum pressure drop of only 59 Pa across the thickest filter, which is comfortable for human breathing. The filter showed negligible loss of PM removal efficiency (<1%) even when completely wet because of the permanent CsW charges, in contrast to the dramatic performance decline exhibited by the N95 commercial filter. Furthermore, our filter fully biodegraded in the composting soil within 4 weeks. Compared to other studies with similar conceptual ideas, in which filters are partially composed of biodegradable materials or which demonstrate only limited performance in terms of potential biopolymer nonwoven applications,^[^ ^6a–d^ ^]^ this filter directly demonstrates biodegradability with high‐level functionality (Movie S1, Supporting Information).

As components of the Janus membrane filter, nanofiber and microfiber PBS mats were first prepared. Accordingly, 11 and 12 wt% PBS solutions were electrospun to produce nanoscale and microscale fibers, respectively, which is attributed to their viscosity difference.^[^ ^7a–c^ ^]^ Details of solution properties and optimum electrospinning conditions are listed in Tables S1 and S2, Supporting Information. Since the as‐spun fibers still contained residual solvents, an additional water coagulation bath was added to the typical electrospinning setup, as shown in **Figure** **2**a. Moreover, the water bath also enabled coagulated pure PBS fiber mats to be collected using a frame, unlike the solid substrate from a conventional setup (Figure 2b).^[^ ^7d^ ^]^ The micro and nanofiber mats showed average fiber diameters of 2.25 and 0.51 µm, and average pore sizes of 13.1 and 3.5 µm, respectively (Figure 2c,d). The difference in the viscosities of the 11 and 12 wt% solutions rapidly increases as the 9:1 chloroform/ethanol solvent rapidly evaporates after its release from the nozzle (Figure S1, Supporting Information).^[^ ^7e,f^ ^]^ Consequently, only a 1 wt% difference in concentration results in a notable change in fiber diameter.

**Figure 2:**
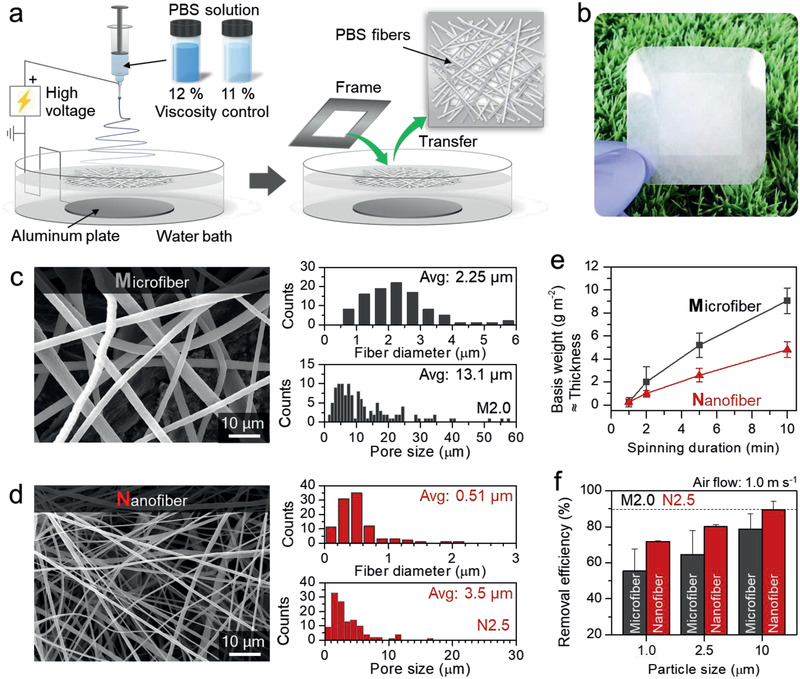
Fabrication process, morphologies, and PM removal performance of PBS microfibers and nanofibers produced from solutions of different concentration. a) Schematically illustrating the electrospinning setup and the production of a PBS fiber mat. b) Fabricated PBS nanofiber mat. c) SEM image of the fabricated microfiber mat and its average fiber diameter and pore size. d) SEM image of the fabricated nanofiber mat and its average fiber diameter and pore size. e) Basis weight (g m^−2^) ≈ thickness of the PBS microfiber (gray) and nanofiber (red) mats prepared for various spinning durations. f) PM removal efficiencies of the microfiber (gray) and nanofiber (red) mats with similar basis weights (2.0 and 2.5 g m^−2^, respectively) for various particle sizes. The removal efficiency was determined at an air velocity of 1.0 m s^−1^.

Before examining filter performance (Figure S2, Supporting Information), an electrospun nonwoven of standard thickness was fabricated in order to reasonably compare the various filters because thickness is a significant influencing factor for filter performance in terms of the pressure differential and filtration efficiency. It is difficult to directly determine the thickness of an electrospun nonwoven, as nonwovens are soft and porous. Fabric thickness is generally proportional to areal density (weight per unit area, the basis weight). Therefore, in this study, we used the basis weight (g m^−2^) as a valid measure of thickness.^[^ ^8a,b^ ^]^ Thickness was controlled by varying the electrospinning time, as shown in Figure 2e. The thickness of the microfiber mat increased to 0.2, 2.0, 5.2, and 9.1 g m^−2^, respectively, as the spinning duration was increased from 1 to 10 min. In the same fashion, the thickness of the nanofiber mat increased to 0.2, 1.0, 2.5, and 4.8 g m^−2^, respectively. The microfiber and nanofiber mats are respectively designated by their thickness values (g m^−2^) as: M0.2, M2.0, M5.2, and M9.1, and N0.2, N1.0, N2.5, and N4.8.

The air pressure differential (Δ*P*) across the sample is an important index of filter performance.^[^ ^9^ ^]^ Breathing through a filter with a high pressure drop is uncomfortable for the user. Naturally, pressure drop was observed to increase with filter thickness, as shown in Figure S3, Supporting Information. The nanofiber mat (N4.8) displayed a higher pressure drop than the microfiber (M5.2) mat at comparable thickness because the nanofiber mat has smaller pores. The pressure drop across the two different types of filter gradually increased from 10^1^ to 10^2^ Pa with air passing through the filters at velocities of between 0.5 and 13.2 m s^−1^. Thickness should be optimized to balance pressure drop and PM removal efficiency; an air velocity of 1.0 m s^−1^ is reasonable because humans exhale at around 1.3 m s^−1^ during mouth breathing.^[^ ^10^ ^]^ In this regard, the pressure drops across M5.2 and N4.8 were acceptable (under 50 Pa) at an air velocity of 1.0 m s^−1^ (Figure S4, Supporting Information). Note that the N95 and the similar Korean filter standard (KF94) masks present pressure drops of 50 to 70 Pa, respectively. Further CsW treatment and micro/nano filter integration can increase air resistance; consequently, to provide a pressure‐drop margin, we analyzed N2.5 and M2.0 before analyzing M5.2 and N4.8.

The PM_1.0_, PM_2.5_, and PM_10_ removal efficiencies of the PBS microfiber and nanofiber mats were investigated in the absence of an electrostatic charge at the target air velocity of 1.0 m s^−1^ (Figure S5, Supporting Information). The PM removal efficiencies were observed to generally increase with increasing thickness and PM size. The removal efficiencies of N2.5 are superior to those of M2.0 due to its smaller pores. M2.0 exhibited removal efficiencies of 55.5%, 64.6%, and 78.8% for PM_1.0_, PM_2.5_, and PM_10_, respectively, while the analogous values for N2.5 are 71.9%, 80.1%, and 89.6% (Figure 2f). We note that the largest difference in the efficiencies of M2.0 and N2.5 was observed for PM_1.0_, which suggests that physical sieving by the microfiber web is effective for microscale PM but not for nanoscale PM (Figure S6, Supporting Information). In addition, both M2.0 and N2.5 presented low PM capturing abilities of less than 90%. Moreover, N2.5 is likely to be more susceptible to dust than M2.0 because dust particles will readily occlude the smaller pores of N2.5. In the absence of an electrostatic charge, physical sieving is limited in its ability to simultaneously achieve the desired pressure drop and removal efficiency because of their trade‐off relationship.

Electrostatic adsorption is the most widely used method for capturing PM in a highly efficient manner.^[^ ^11a–c^ ^]^ In general, an electrostatic charge is forcibly applied to a nonwoven filter by a high energy electric field; however, this electrostatic charge readily dissipates under humid conditions, resulting in the loss of PM capturing capacity.^[^ ^4e,f^ ^]^ As a bio‐based material for electrostatic filtration, we introduced 200 nm long and 40 nm wide CsWs; these nanowhiskers contain permanent cationic charges due to their ammonium and polar amide groups. The available positive charge on the surface of a CsW is represented by its zeta potential (ZP); CsWs were dispersed in water at pH 4.8, where they were found to have a ZP of +49.8 mV (Figure S7, Supporting Information).

CsW‐coated PBS microfibers (ChMs) and nanofibers (ChNs) were prepared by simple dip‐coating in a 0.2 wt% aqueous CsW dispersion, which is an appropriate concentration for attaching the maximum amount of CsWs onto the PBS fiber surface, as shown in **Figure** **3**a and Figure S8, Supporting Information. The nitrogen energy dispersive X‐Ray spectroscopy (EDS) map reveals that the surfaces of the PBS fibers are uniformly coated with CsW particles, which is also evident in the scanning electron microscopy (SEM) image (Figure 3b; Figure S9, Supporting Information). Moreover, this coating method enables charged nanomaterials to sophisticatedly enwrap the fiber surfaces, which maximizes the electrostatic PM removal ability (Figure S10, Supporting Information).

**Figure 3:**
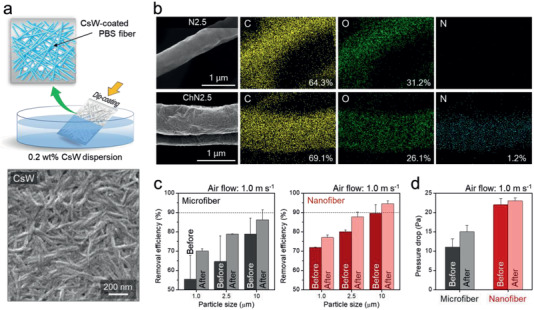
CsW‐coated filter and its filtration performance. a) PBS fiber mat dip‐coated using the CsW dispersion, and an SEM image of CsWs ≈200 nm in length. b) EDS maps used to investigate pure PBS fibers and a uniformly well‐coated CsW‐PBS fiber. c) Effect of CsW on the removal efficiency for PM_1.0_, PM_2.5_, and PM_10_ using the microfiber (left graph) and nanofiber (right graph) mats, before (dark gray: M2.0 and dark red: N2.5) and after (light gray: ChM2.0 and light red: ChN2.5) CsW‐coating. d) Pressure differential before and after coating the microfiber (gray: ChM2.0) and nanofiber (red: ChN2.5) mats with CsW. The removal efficiency and pressure drop were determined at an air velocity of 1.0 m s^−1^.

The PM removal efficiencies of ChM and ChN were investigated (Figure 3c). M2.0 and N2.5 were coated with CsW to produce ChM2.0 and ChN2.5, respectively. The removal efficiencies of ChM2.0 for PM_1.0_, PM_2.5_, and PM_10_ were determined to be 70.1%, 78.8%, and 86.3%, respectively, while the analogous values for ChN2.5 are 77.0%, 87.7%, and 94.6%, respectively. The CsW coating greatly improves the removal efficiencies of M2.0 and N2.5, with the more remarkable effect observed for the somewhat smaller PM. In particular, the chitosan nanowhiskers improve the PM_0.5_ and PM_1.0_ removal efficiencies of M2.0 by 15% and 13%, respectively (Figure S11, Supporting Information). While M2.0 poorly excludes the smaller PM_1.0_ due to its relatively wide inter‐fibril spacing (Figure 2c), ChM2.0 adsorbed PM_1.0_ because the cations and amides in the CsWs interact through ion–ion, dipole–ion, and dipole–dipole interactions with dust. As a result of their CsW coatings, the PM removal efficiencies of ChM2.0 and ChN2.5 are as high as those of the thicker M5.2 and N4.8 (Table S3, Supporting Information).

Interestingly, the CsW coating hardly affected the pressure drop, despite the large improvement in PM removal efficiency. The drops in pressure across ChM2.0 and ChN2.5 increased slightly to 15 and 23 Pa, which are almost half of the increments observed for M5.2 and N4.8 (Figure 3d; Table S3, Supporting Information). Thus, coating with a bio‐based material is a suitable way of satisfying the two essential filter performance requirements; namely, PM removal efficiency and the air pressure differential, which are mutually exclusive. Nevertheless, the PM_1.0_ and PM_2.5_ removal efficiencies of both ChM2.0 and ChN2.5 were below 90%; clearly, this performance needs to be improved.

An integrated filter system composed of multiple membranes with gradually varying fiber diameters and pore sizes can solve the abovementioned problems.^[^ ^12a–f^ ^]^ An integrated air filter has the advantage of two different nanofiber and microfiber webs. In this regard, ChM and ChN were simply stacked to produce integrated filters (Int‐MNs). For example, Int‐MN4.5 was prepared using ChM2.0 and ChN2.5, and its performance was compared with that of ChN4.8 and ChM5.2 with similar areal densities (i.e., thicknesses). In PM removal efficiency experiments, the microfiber side of Int‐MN4.5 was exposed to a dusty chamber because the microfiber side is somewhat more resistant to blockage than the nanofiber side. As shown in **Figure** **4**a, Int‐MN4.5 exhibited a superior PM removal efficiency and pressure differential than the two single‐component filters, with a pressure drop of 37 Pa, which is similar to that of ChM5.2 and much lower than that of ChN4.8. Furthermore, Int‐MN4.5 delivered a PM_1.0_ removal efficiency of 91% (Figure 4b). On the other hand, ChM5.2 did not present such high PM_1.0_ removal efficiencies because its pores are larger than those of Int‐MN4.5.

**Figure 4:**
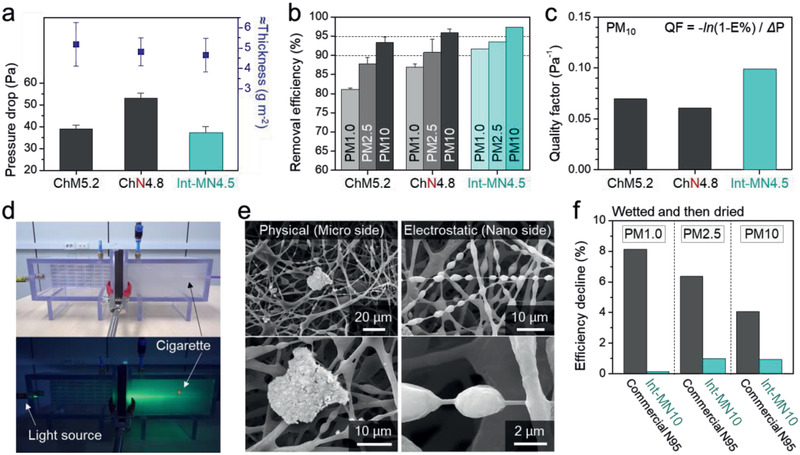
Integrated filters with superior characteristics, such as low pressure drops, high‐efficiencies, and permanent charges. Comparing a) pressure drops, b) PM removal efficiencies, and c) quality factors, of single‐layered and integrated filters. d) Successfully blocking the PM from a cigarette source as demonstrated by Tyndall light scattering (Movie S2, Supporting Information). e) SEM images showing the physical and electrostatic PM capturing abilities of the integrated filter. f) Efficiency declines of commercial N95 and the integrated filter after exposure to moisture.

The quality factor (QF) of Int‐MN4.5 also confirms its superior performance. QF is a representative tool for assessing overall filter performance and is determined by the balance between efficiency and pressure drop. Int‐MN4.5 displayed a higher QF than ChM5.2 and ChN4.8 (Figure 4c); thus, the pore‐size distribution gradient and the sufficient amount of coated chitosan of Int‐MN4.5 contribute effectively to its low pressure drop and high PM capturing efficiency.

The effective PM blocking performance of Int‐MN4.5 is visually shown in Figure 4d and Movie S2, Supporting Information. A serious level of PM was generated by burning a cigarette; the filter, which was positioned between the PM source and another empty box, was visually confirmed to completely block the generated smoke from proceeding into the empty box through the Tyndall effect. The microfiber side that directly faces the smoky chamber sieves relatively large PM, after which the nanofiber side adsorbs relatively small PM through electrostatic interactions (Figure 4e).

Int‐MN4.5 has an additional pressure drop margin compared to the N95 and KF94 mask filters. To improve removal efficiency, Int‐MN7.7 and Int‐MN10 were also prepared in the same manner (Table S4, Supporting Information). Int‐MN10 delivered a somewhat higher removal efficiency than the N95 mask filter, while providing a comparable drop in air pressure of 59 Pa (Figure S12, Supporting Information); its PM removal efficiency for PM_1.0_, PM_2.5_, and PM_10_ were 97.5%, 98.3%, and 99.24%, respectively. Nevertheless, on the basis of QF, Int‐MN4.5 is a sufficiently effective filter as it delivers a PM_1.0_ removal efficiency of 91% (Table S4, Supporting Information).

As mentioned above, conventional electrostatic filtration systems can suffer performance losses at any time when used in ambient humidity, such as wet weather or exhaled moisture. Hence, the US Centers for Disease Control and Prevention (CDC) strongly recommends restricting the use of an N95 mask to 1 day or replacing it after exposure to moisture. As an N95 mask is unsuitable for reuse once contaminated with viruses or bacteria, a folk remedy involves fully spraying an aqueous ethanol solution onto the filter as a disinfectant; however, this moisture‐rich treatment removes the electrostatic charge of the filter. Of note, our filter is outstandingly moisture‐resistant, as shown in Figure 4f. As an objective comparison, we tested Int‐MN10 and a commercial N95 mask filter, which have similar removal efficiencies. Both filters were completely wetted by spraying with water, and then dried. Int‐MN10 showed an average decline in efficiency of less than 1%, despite being thinner than the N95 mask filter (Figures S12 and S13, Supporting Information), whose removal efficiency for PM_1.0_, which is most affected by electrostatics, declined by more than 8%. Moreover, even after repeatedly being subjected to 45 wet–dry cycles, Int‐MN10 still maintained a PM_1.0_ removal efficiency of over 95% (Figure S14, Supporting Information). Given that the wet–dry‐cycle testing conditions used are extremely harsh compared to ambient moisture, this performance drop is negligible. Therefore, it is possible to prevent the natural loss of electric charge under ambient moisture, which enables longer storage and multiple mask use. In addition, our filter can be treated with an aqueous ethanol solution.

It is particularly noteworthy that the highly efficient filter in this study is made entirely of biodegradable materials, which is a novelty. The enormous volume of masks currently in use will clearly lead to a massive waste crisis in the near future. At a temperature of 50 °C, ChN2.5 was completely decomposed within 7 h by the lipase enzyme from *Thermomyces lanuginosus*, as shown in **Figure** **5**a and Movie S3, Supporting Information. Moreover, it also fully decomposed in composting soil at room temperature within 4 weeks (Figure 5b; Figure S15, Supporting Information). Therefore, biodegradable materials, especially CsW and PBS, provide obvious mask waste‐disposal solutions.

**Figure 5:**
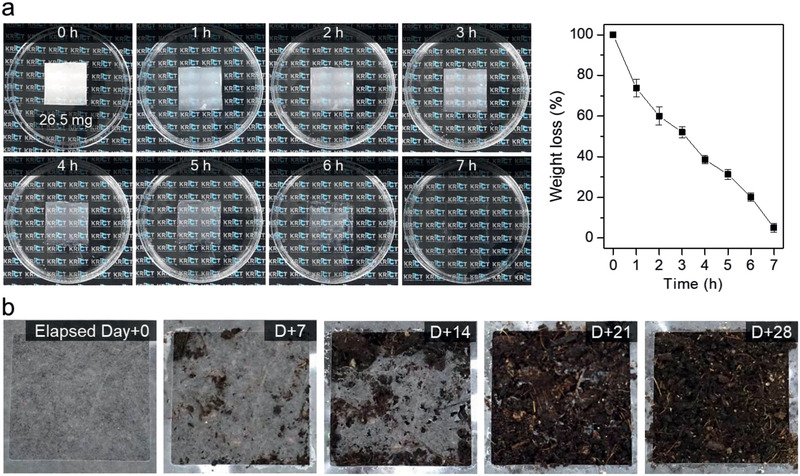
Biodegradability of the developed filter. a) Time‐dependent enzymatic degradation images of the CsW‐coated PBS filter and the corresponding weight loss as a function of time (enzyme: lipase from *Thermomyces lanuginosus*; Movie S3, Supporting Information). b) Images showing the degradation of the CsW‐coated PBS filter in the composting soil over time.

The functions of chitosan enable multiple and long‐term mask‐filter usage, and chitosan is well known to have antibacterial properties.^[^ ^13a,b^ ^]^ We investigated whether or not the chitosan nanomaterial also adsorbs viruses, which can become attached to surfaces that are appropriately ionically charged.^[^ ^13c–e^ ^]^ In our model study, we sprayed an aqueous solution containing the *Escherichia coli* bacteriophage Phi X174 virus into a closed chamber, and then the virus‐containing air was passed through the CsW‐coated PBS fiber non‐woven web as in the PM removal test. As a result, a viral cluster ≈50 nm in size became bound to the CsW‐coated PBS fiber (Figure S16a, Supporting Information). To determine how CsW binds to the virus, the aqueous virus solution was sprayed onto a CsW‐coated plastic film surface, after which it was vigorously washed with water; the virus was still attached to the surface following washing (Figure S16b,c, Supporting Information). This does not mean that the filter completely prevented virus penetration; however, the CsW coating seems to effectively adsorb viruses. As this experiment only explored whether or not viral attachment is possible, more in‐depth studies are required in the future.

In summary, a biodegradable and highly efficient mask filter was manufactured using PBS and CsW. The permanent ionic charges provided by the easy and simple CsW coating process not only engender the PBS microfibers and nanofibers with electrostatic PM adsorption capabilities, but they also prevent charge dissipation under ambient humidity. One of the fabricated filters (Int‐MN10) removed more than 97% of PM greater than 1 µm in size and delivered an acceptable air pressure drop of 59 Pa. These outcomes reveal that we successfully developed a high‐level PM‐capturing mask filter that provides a comfortable breathing environment for the user and whose performance does not decline. Furthermore, unlike commercial disposable face masks, the developed filter is sustainable; it fully biodegrades within 1 month in the composting soil. The antibacterial and virus‐blocking properties of chitosan are also expected to be exhibited by the developed filter. This filter is expected to be a great future practical alternative to conventional disposable filters.

## Experimental Section

Experimental details are given in the Supporting Information.

## Conflict of Interest

The authors declare no conflict of interest.

## Supporting information

Supporting Information

Supplemental Movie 1

Supplemental Movie 2

Supplemental Movie 3
