# Supplementary material for: Biodegradable, Efficient, and Breathable Multi‐Use Face Mask Filter
Source: Adv Sci (Weinh). 2021 Jan 29;8(6):2003155. doi: 10.1002/advs.202003155 (PMC7967051; doi:10.1002/advs.202003155)
Supplement: Supplementary file 1 — Supporting Information [file ADVS-8-2003155-s001.pdf]

WILEY-VCH

© 2020 Wiley-VCH GmbH

ADVANCED SCIENCE
Open Access

Supporting Information

for *Adv. Sci.,* DOI: 10.1002/advs.202003155

Biodegradable, Efficient, and Breathable Multi-Use Face Mask Filter

Sejin Choi, Hyeonyeol Jeon, Min Jang, Hyeri Kim, Giyoung Shin, Jun Mo Koo, Minkyung Lee, Hye Kyeong Sung, Youngho Eom, Ho-Sung Yang, Jonggeon Jegal, Jeyoung Park, Dongyeop X. Oh*, Sung Yeon Hwang**

Supporting Information

Biodegradable, Efficient, and Breathable Multi-Use Face Mask Filter

Sejin Choi, Hyeonyeol Jeon, Min Jang, Hyeri Kim, Giyoung Shin, Jun Mo Koo, Minkyung Lee, Hye Kyeong Sung, Youngho Eom, Ho-Sung Yang, Jonggeon Jegal, Jeyoung Park, Dongyeop X. Oh*, Sung Yeon Hwang**

Experimental section

Materials: Poly(butylene succinate) (PBS, M_n = 72,300 g mol^{-1}) was prepared as previously described.[S1] α-Chitin from shrimp shells (practical grade, powder) and sodium hydroxide (NaOH, 97%) were purchased from Sigma-Aldrich Co. (USA). Chloroform (99.8%, HPLC grade) was purchased from Avantor Co. (USA), and hydrochloric acid (HCl, 37%) was purchased from Daejung Co. (Republic of Korea).

Solution preparation and PBS electrospinning: Two PBS solutions (11 and 12 wt%) were prepared by in a 9:1 (w/w) mixture of chloroform and ethanol (Table S1). The prepared 12- and 11-wt% PBS solutions were passed through 25 and 26 gauge metal nozzles, respectively, at a flow rate of 4 mL h^{-1} under syringe-pump control (EP120, NanoNC Co., Republic of Korea). A voltage of 7.5 kV was applied to each nozzle tip with a high-voltage power supply (B130, KSC Co., Republic of Korea). The distance between the nozzle tip and the water-bath-grounded collector electrode was fixed at 20 cm (Table S2); this collector electrode consisted of a round aluminum plate and a round glass petri dish, which were 8 and 20 cm wide, respectively. The round metal plate was placed inside the bath and filled with tap water to a depth of 1.5 cm. The electrospun nanofiber mat collected on the surface of the bath and was transferred to a square frame made from a 100-μm-thick polyethylene terephthalate (PET) film cut into a square ring with inner and outer lengths of 3 and 10 cm, respectively.

Preparing chitosan nanowhiskers (CsWs) and the dip-coating method: α-Chitin powder (5 g) was hydrolyzed in 150 mL of 3 M HCl solution at 120 ºC for 3 h and then fully washed with deionized water to remove HCl. The hydrolyzed chitin was neutralized using dilute NaOH solution, dialyzed to remove all residual salts, and freeze-dried for storage. The hydrolyzed chitin (3 g) was subsequently deacetylated by heating in 30% NaOH solution (100 mL) at 80 ºC for 6 h. The product was thoroughly washed with deionized water to remove excess NaOH, neutralized to pH 7 with dilute HCl, dialyzed, and freeze-dried. The synthesized CsWs were then re-dispersed in deionized water with an ultrasonic processor (Sonics, Vibra-Cell, USA). The dispersion was ultrasonicated for 10 min (pulse on and off for 10 and 5 s, respectively) at an amplitude of 40%. The final pH was adjusted to four by adding a few drops of 0.1 M HCl. The prepared CsWs were dispersed in deionized water to a concentration of 0.2 wt%. The pre-prepared electrospun PBS fiber mats were immersed sequentially in the CsW dispersion with constant stirring at room temperature until the mats were fully wet. The resulting CsW-coated PBS fiber mats were dried at room temperature for 24 h.

PM removal efficiency and pressure drop measurement: Particulate matter (PM) was generated by burning paper pieces. The paper pieces were made using a paper punch with fixed size of 5 mm in diameter, then burned in a closed PM source chamber, as shown in Figure S2. The generating amount of PM was controlled by the number of burned paper pieces. The generated PMs were blown by an air compressor and the air velocity crossing the filter was adjusted by the pressure of compressed air. The number of PM was counted by a particle counter (DT-9881M, CEM Instrument Co., China) and the removal efficiency was calculated by comparing the number of particles detected with and without filters. Unless mentioned, the relative humidity of the efficiency test was around 15%. The pressure drop (ΔP) was measured by a pressure and flow meter (DT-8920, CEM Instrument Co., China).

Virus adsorption test: *Escherichia coli bacteriophage Phi X174* was purchased from American Type Culture Collection (ATCC, USA). The virus of 0.5 mL was added to a conical tube, and subsequently diluted 10-fold in distilled water. The virus solution was sprayed into a closed PM source chamber using a humidifier, then blown by compressed air to the filter.

Biodegradation test: A CsW-PBS microfiber mat (~26.5 mg) was prepared and then enzymatically degraded in 0.1 M phosphate buffer solution at pH 6.5 and 50 ºC with lipase from *thermomyces lanuginosus*. The weight of the filter was measured in 1-h intervals. Landfill degradation was carried out by burying the filter in the soil to a depth of 3 cm. The degradation images were captured using a digital camera (DSC-RX100M4, Sony Co., Japan) at one-week intervals. The soil on the filter was carefully swept off to record the image, and the filter was then re-covered with soil. The aerobic PBS biodegradation results, determined through the generation of carbon dioxide (based on ISO 14855-1), were provided by Organic Waste Systems (OWS Co., Gent, Belgium), a biodegradability certification company.

General characterization: Air-flow velocity was measured using a digital air velocity meter (TPI-575, Test Products International Inc., OR, USA). The changes in the viscosity of the electrospinning solution over time were measured using a rotational rheometer (MCR 302, Anton Paar GmbH Co., Austria). SEM images and EDS maps were acquired on a field-emission scanning electron microscope (Tescan MIRA3, Czech Republic) equipped with energy dispersive X-ray spectroscopy capabilities (NORAN System 7, Thermo Fisher Scientific Inc., MA, USA). Zeta potentials (ZPs) were measured using a Zetasizer Nano Z instrument (Malvern Panalytical Ltd., UK). The concentration-dependent optical transmittance of the aqueous CsW dispersion was measured using a UV-VIS spectrophotometer (UV-2600, Shimadzu. Co., Japan).

Table S1. Typical properties of solvents.

	Dielectric constant[a)]	Relative polarity[b)]	Vapor pressure [hPa][b)]	Boiling point [ºC][b)]	PBS solubility[c)]
Chloroform	4.81	0.259	210	61.2	High
Ethanol	24.6	0.654	59	78.5	Poor
Water[d)]	80.1	1.000	17.5	100	Insoluble

[a)] These data were obtained from CRC Handbook of Chemistry and Physics, 87th edition. [b)] These data were obtained from Solvents and Solvent Effects in Organic Chemistry, 3rd edition. [c)] PBS solubility was determined by observing solution transparency with the naked eye after stirring for 24 h. [d)] Water is listed for intuitive comparison purposes.

Table S2. Electrospinning conditions for preparing PBS microfibers and nanofibers.

	Solution concentration	Nozzle size [G]	Distance [cm]	Flow rate [mL h^{-1}]	Applied voltage [kV]
Nanofiber	11 wt%	26	20	4	7.5
Microfiber	12 wt%	25	20	4	7.5

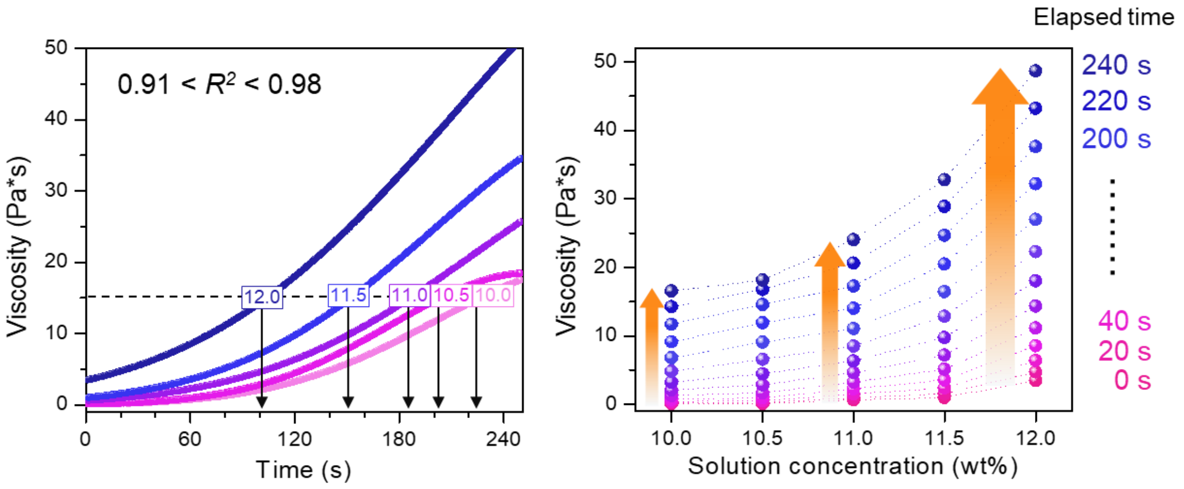

Figure S1. Solution viscosities as functions of elapsed time and solution concentration.

Figure S1 shows how the viscosities of various PBS solutions change with time in an open system. The viscosities of the solutions (10.0, 10.5, 11.0, 11.5, and 12.0 wt%) increased rapidly with elapsed time. However, the viscosity change in going from 11 to 12 wt% was much more remarkable than that observed in going from 10 to 11 wt% due to the extremely fast evaporation rate of the chloroform and ethanol mixture used as the solvent. Significant differences in solution viscosity induce noticeable differences in the fiber elongation rate. Therefore, microfibers and nanofibers were successfully produced with only a 1 wt% difference in solution concentration.

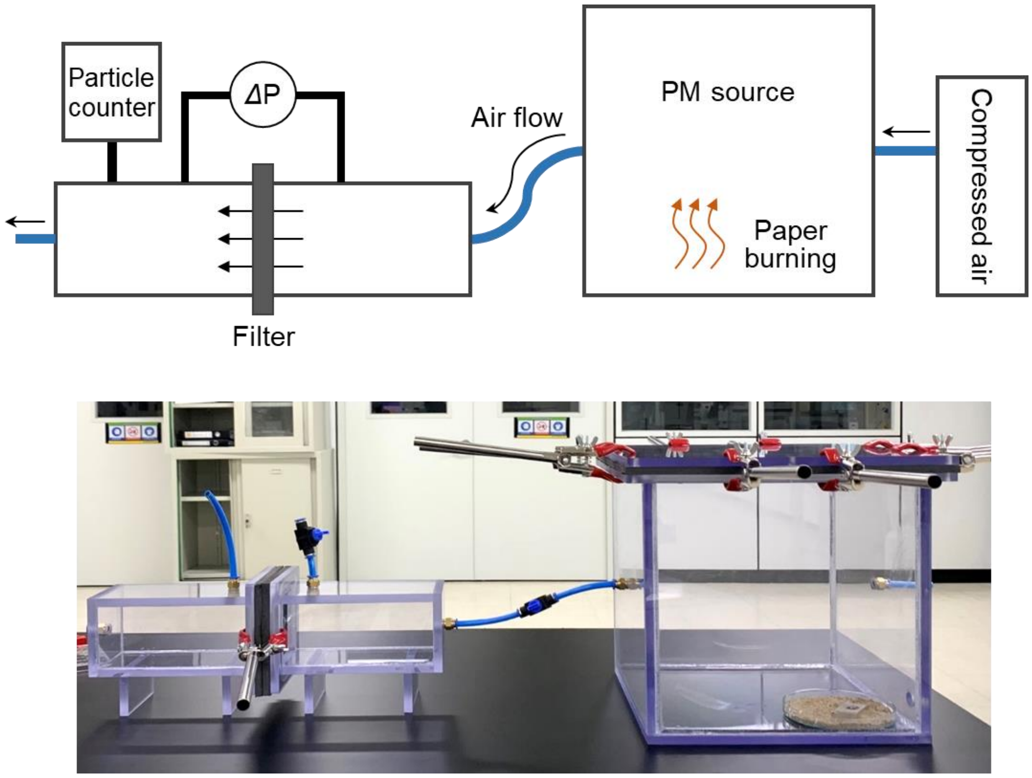

Figure S2. Setup used to measure pressure drop and removal efficiency.

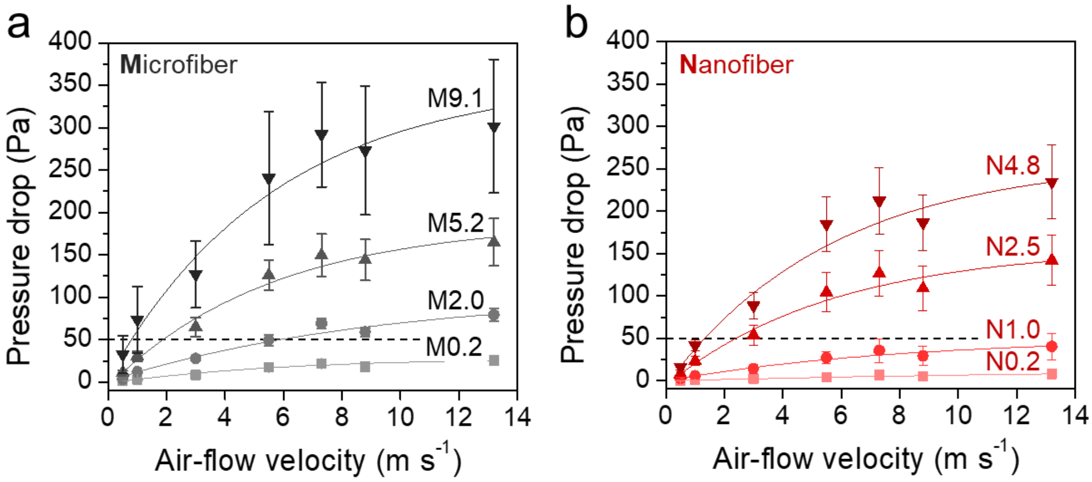

Figure S3. Pressure drops as functions of air-flow velocity. a) Microfiber mats and b) nanofiber mats with various thicknesses.

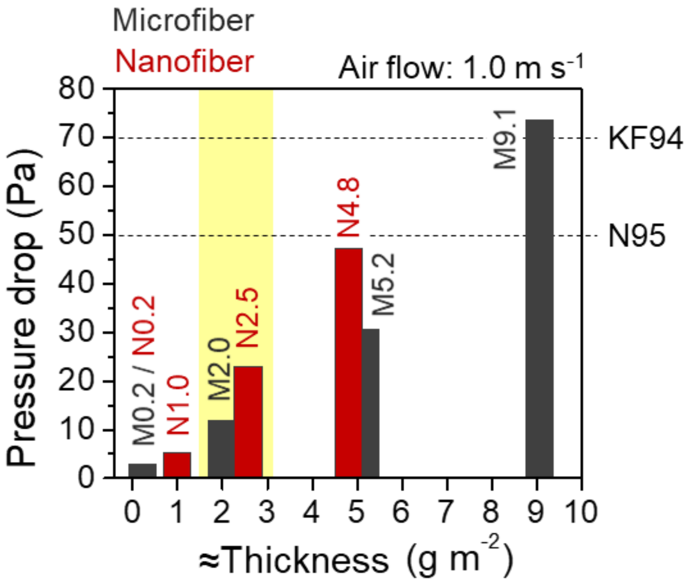

Figure S4. Comparing pressure drops across microfiber (dark gray) and nanofiber (red) mats with various thicknesses at an air-flow velocity of 1.0 m s^{-1}.

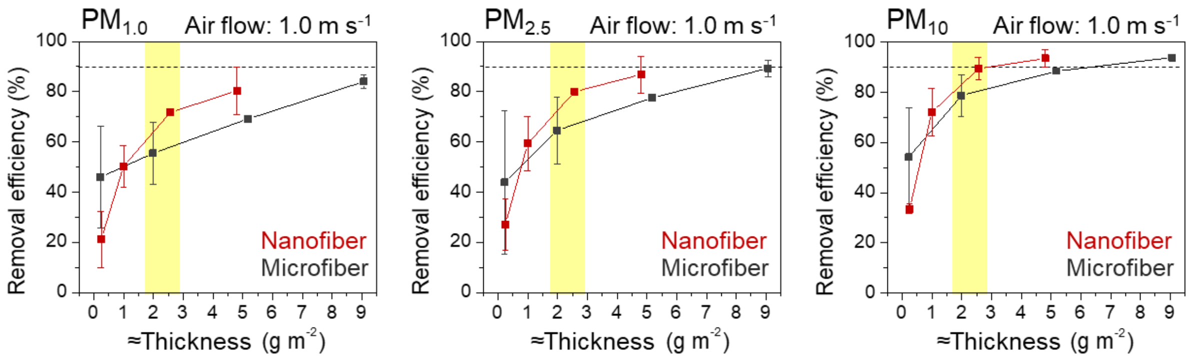

Figure S5. Removal efficiencies of microfiber and nanofiber mats for variously sized PM as functions of thickness.

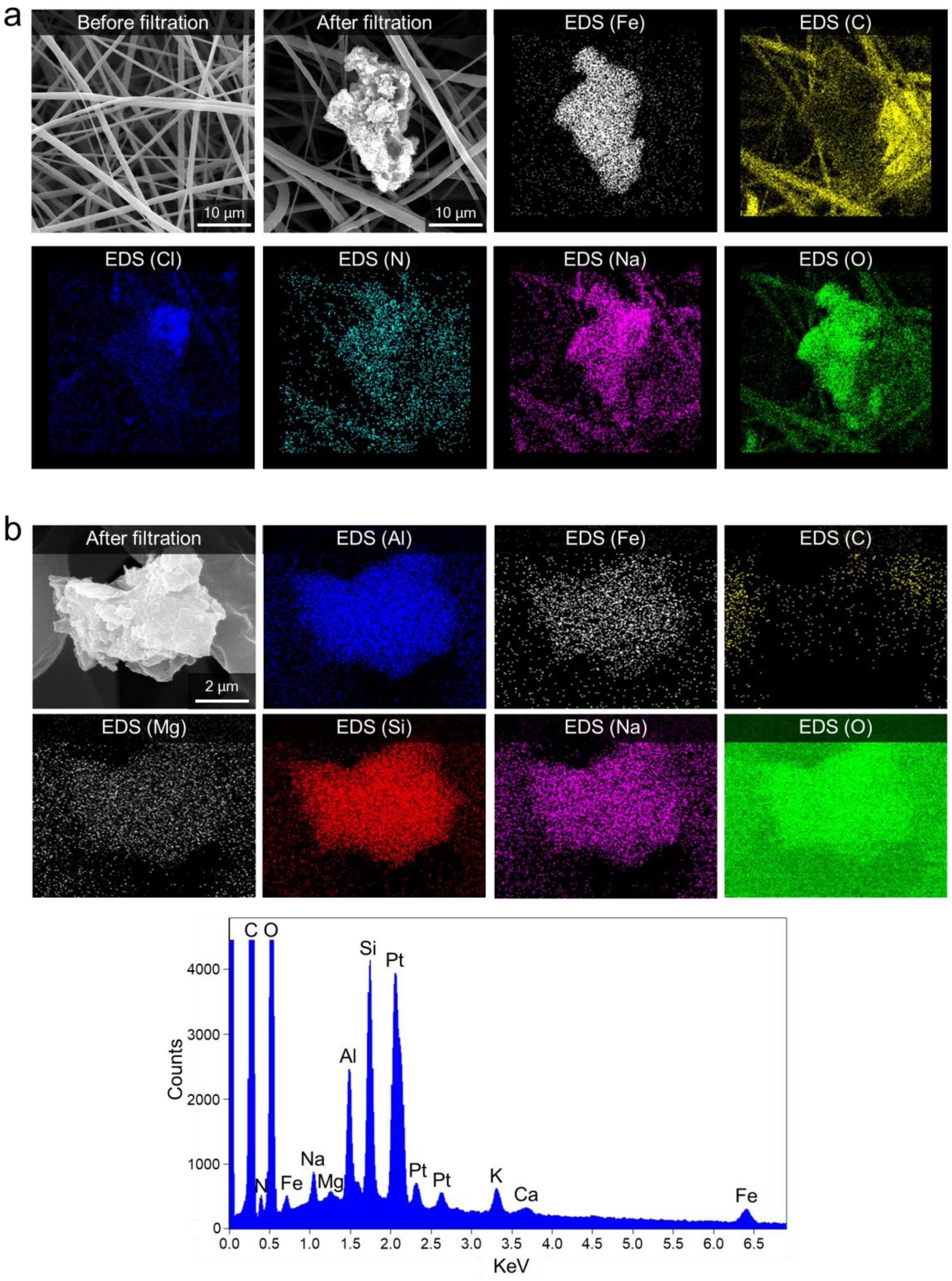

Figure S6. a) SEM images of the as-spun PBS filter before and after filtration, and EDS elemental maps of a physically captured iron-based particle. b) A SEM image and EDS elemental maps of a physically captured metal oxide particle, and the corresponding EDS spectrum.

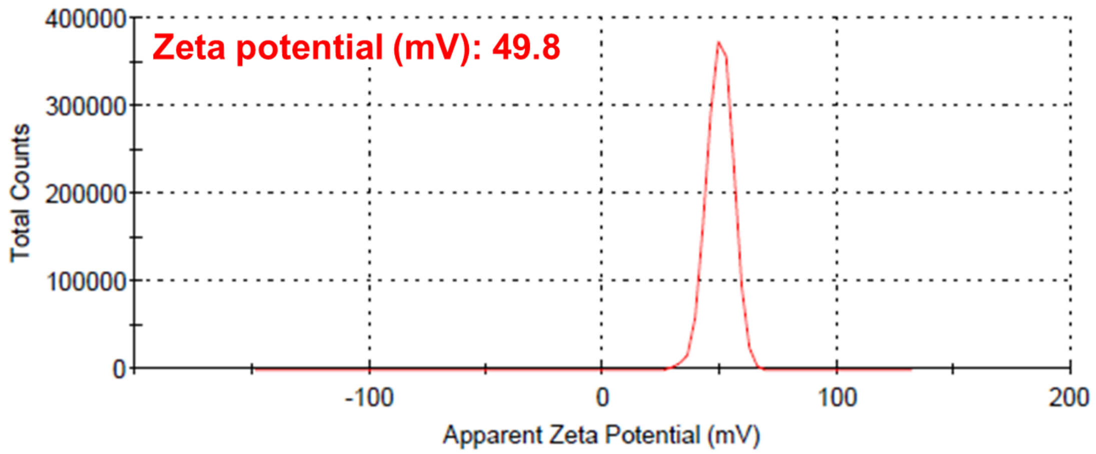

Figure S7. Determining the zeta potential of chitosan nanowhiskers at pH 4.8.

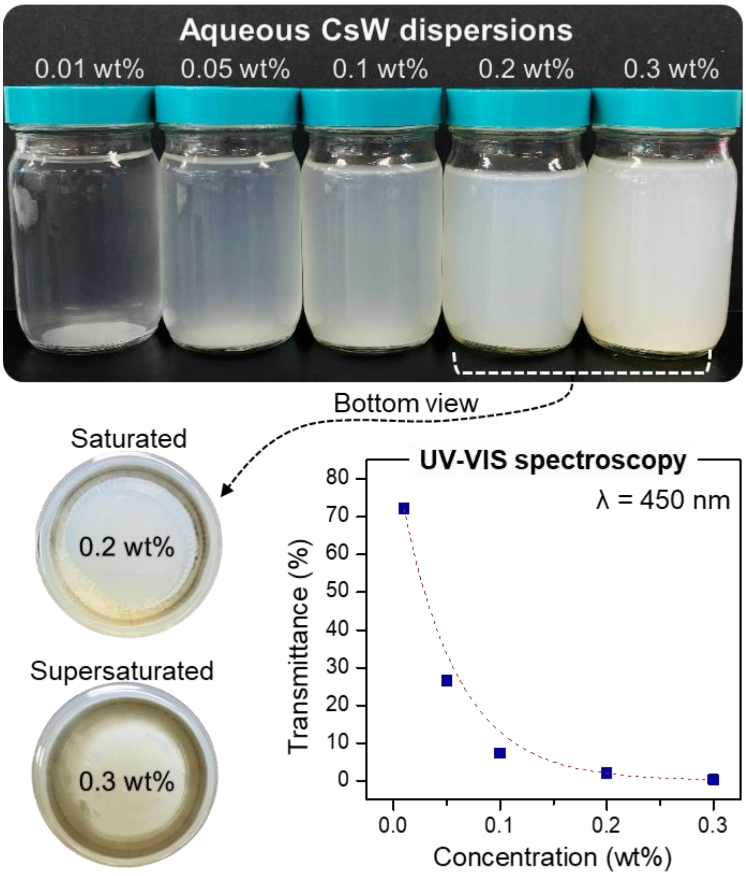

Figure S8. Transmittances of CsW dispersions with various concentrations.

The excessively dispersed CsWs began to agglomerate at a dispersion concentration of 0.3 wt%. Moreover, the UV-VIS transmittance at 450 nm becomes saturated at a concentration of 0.2 wt%. Therefore, 0.2 wt% was the maximum concentration used to prepare a well-dispersed solution for coating the maximum amount of CsWs on the PBS fiber surface.

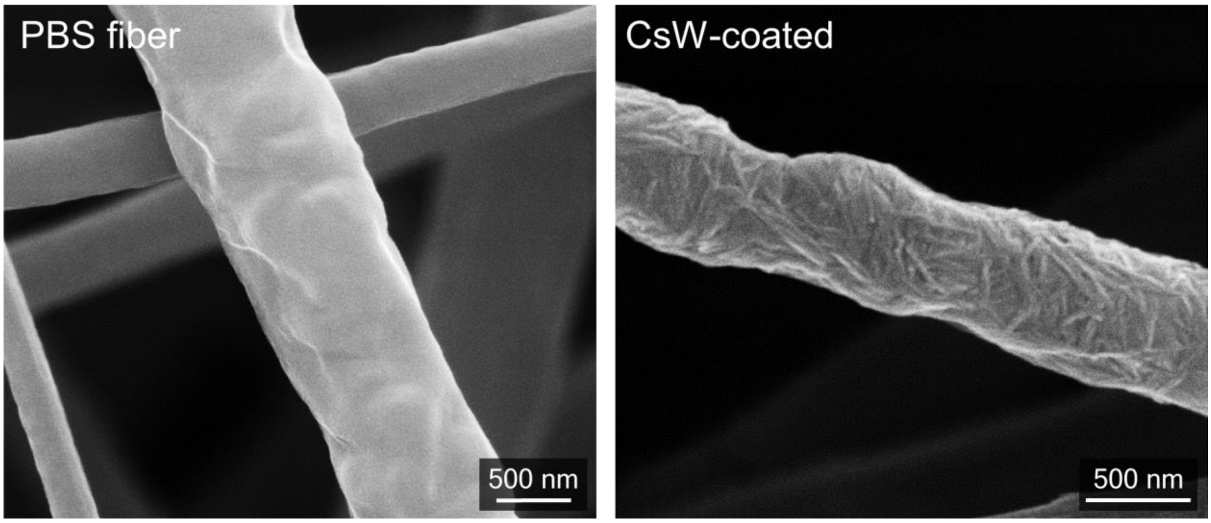

Figure S9. SEM images of a non-coated pure PBS fiber (left) and a CsW-coated fiber (right).

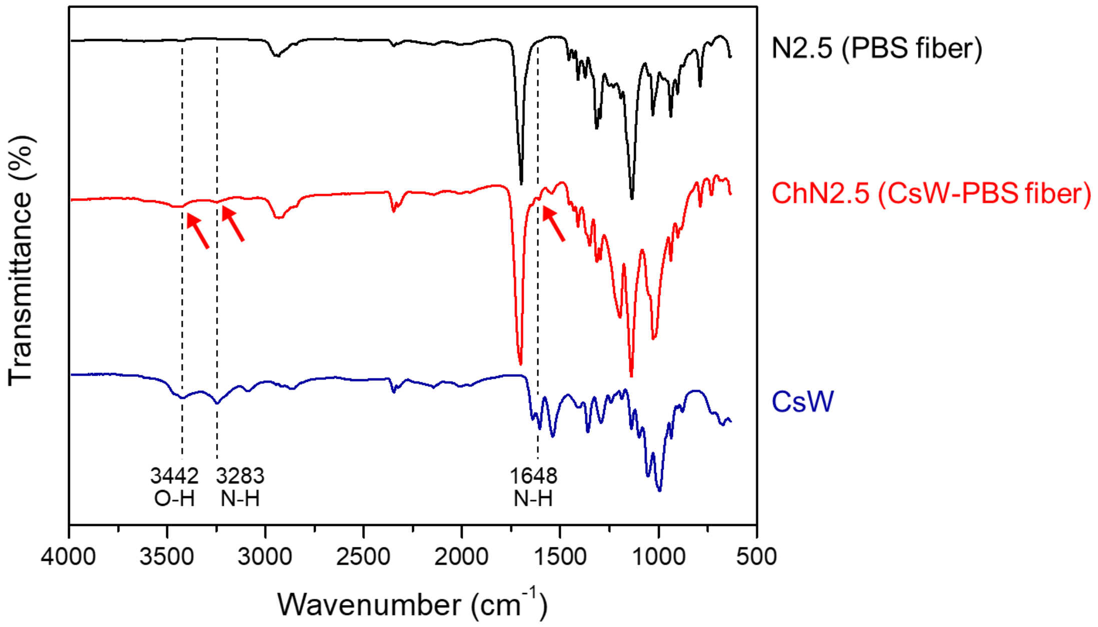

Figure S10. ATR-FTIR spectra of pure PBS fiber, CsW-PBS fiber, and CsW powder.

The polar groups of chitosan were successfully attached to the CsW-coated PBS fibers (ChN2.5) by simple dip-coating.

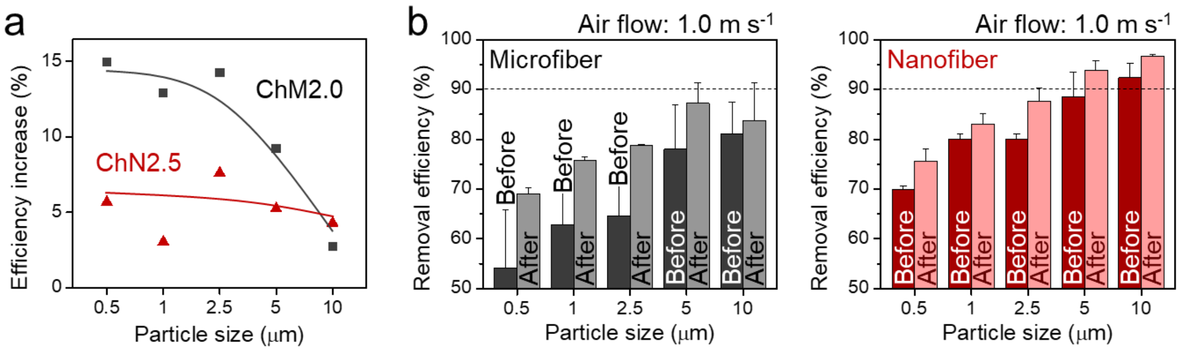

Figure S11. a) Changes in efficiency for ChM2.0 and ChN2.5 calculated from b) removal efficiencies according to particle size, before and after coating the microfibers and nanofibers with CsW.

Table S3. Comparing the PM removal efficiencies and pressure drops of various filters.

	M2.0	ChM2.0	M5.2	N2.5	ChN2.5	N4.8
$E_{1.0}$ [%][a)]	55.5	70.1	69.2	71.9	77.1	80.4
$E_{2.5}$ [%][b)]	64.6	78.8	77.6	80.1	87.7	86.8
E_{10} (%)[c)]	78.8	86.3	88.5	89.6	94.6	93.6
ΔP (Pa)[d)]	11	15	31	22	23	47

[a)] Removal efficiency for $PM_{1.0}$. [b)] Removal efficiency for $PM_{2.5}$. [c)] Removal efficiency for PM_{10}. [d)] Pressure drop at an air-flow velocity of 1.0 m s^{-1}.

Table S4. Performance of the developed filters.

	$E_{1.0}$ (%)	$E_{2.5}$ (%)	E_{10} (%)	ΔP (Pa)	$QF_{1.0}$ [Pa^{-1}][a)]	$QF_{2.5}$ [Pa^{-1}][b)]	QF_{10} [Pa^{-1}][c)]	Basis weight [$g\ m^{-2}$]
ChM5.2	81.15	87.75	93.36	39	0.043	0.054	0.070	5.18
ChM4.8	86.94	90.76	95.95	53	0.038	0.045	0.060	4.81
Int-MN4.5	91.71	93.47	97.37	37	0.067	0.074	0.098	4.56
Int-MN7.7	94.66	96.60	98.73	48	0.061	0.070	0.091	7.75
Int-MN10	97.50	98.30	99.24	59	0.063	0.069	0.083	9.99

[a)] Quality factor for $PM_{1.0}$. [b)] Quality factor for $PM_{2.5}$. [c)] Quality factor for PM_{10}.

*$QF = -ln(1-E\%) / \Delta P$

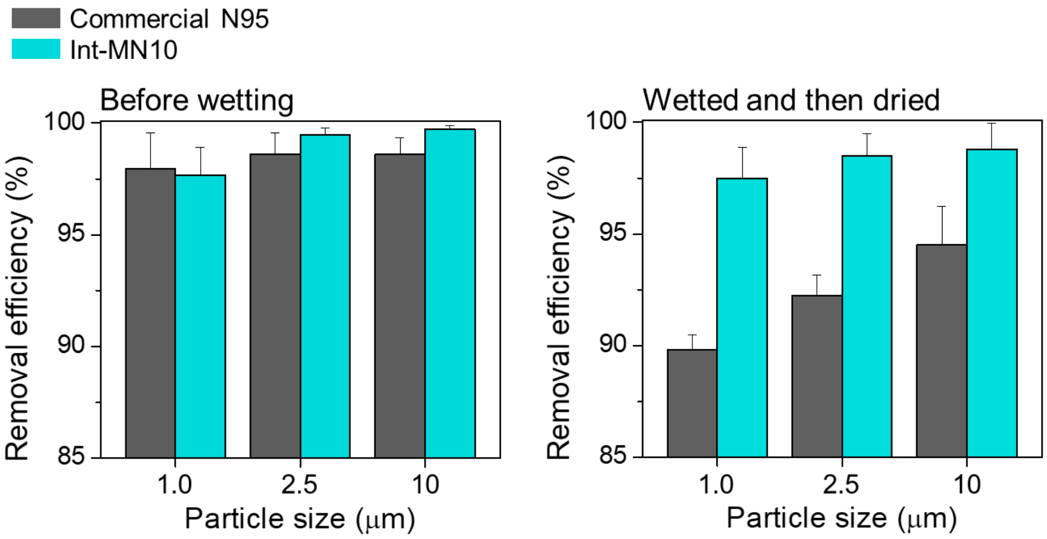

Figure S12. PM removal efficiencies of commercial N95 and the integrated filter for various particle sizes, before and after exposure to moisture.

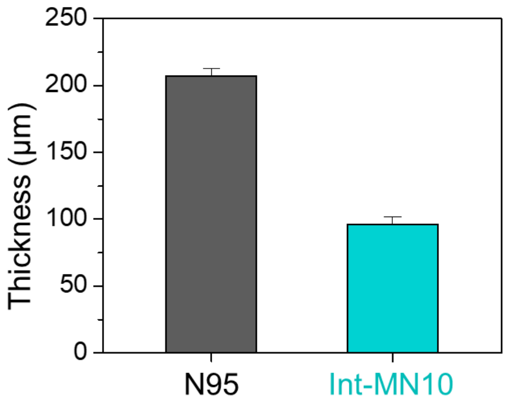

Figure S13. Average thicknesses of the commercial N95 and integrated filters.

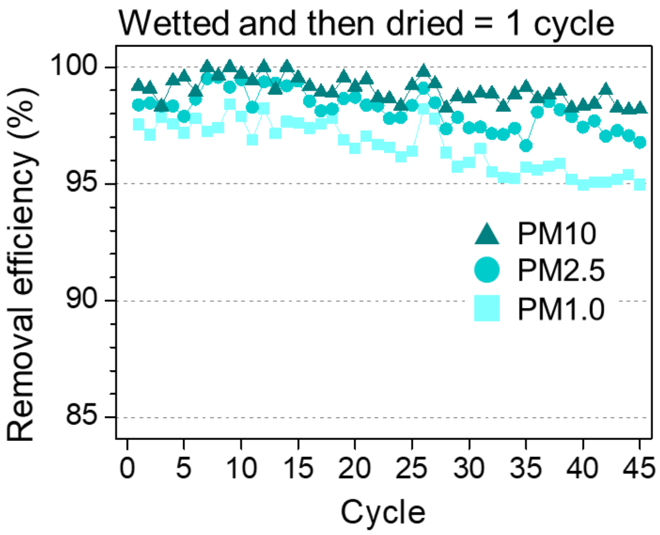

Figure S14. Removal efficiencies of the integrated filter (Int-MN10) over 45 repeated wet-dry cycles.

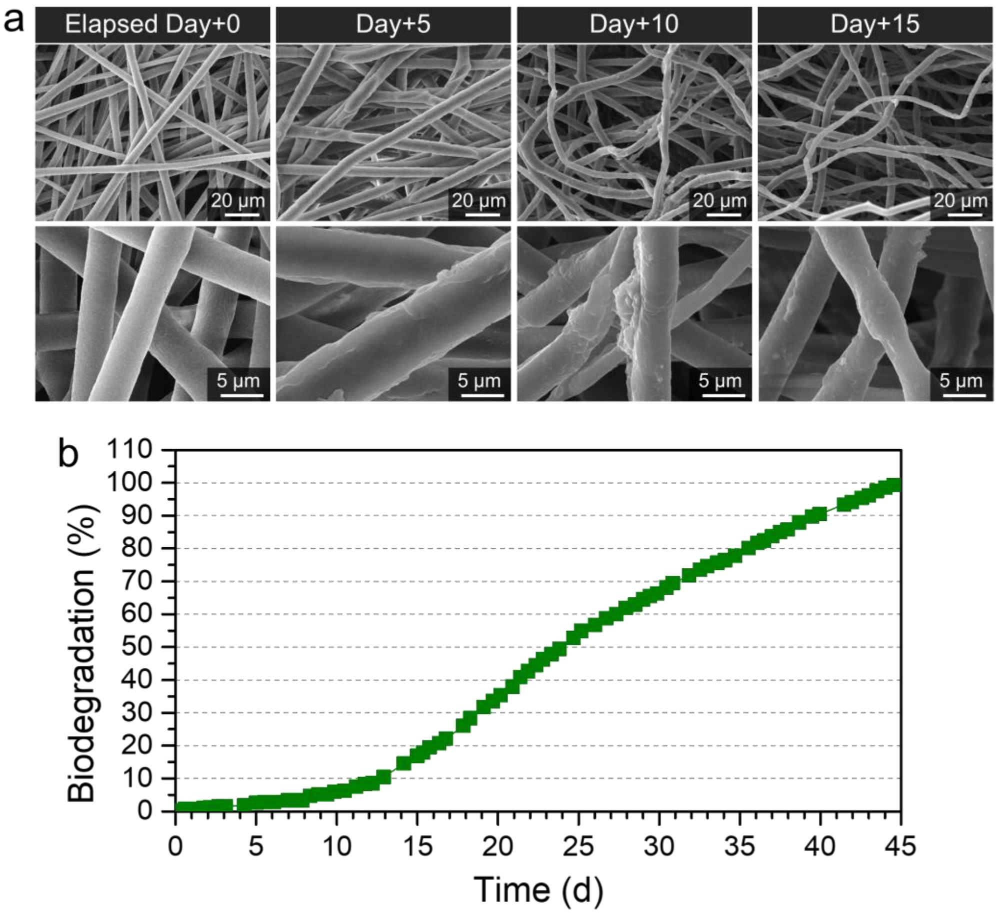

Figure S15. a) SEM images showing the degradation of the CsW-coated PBS filter in composting soil over fifteen days. b) Biodegradation of PBS under composting conditions based on the ISO 14855-1 method.

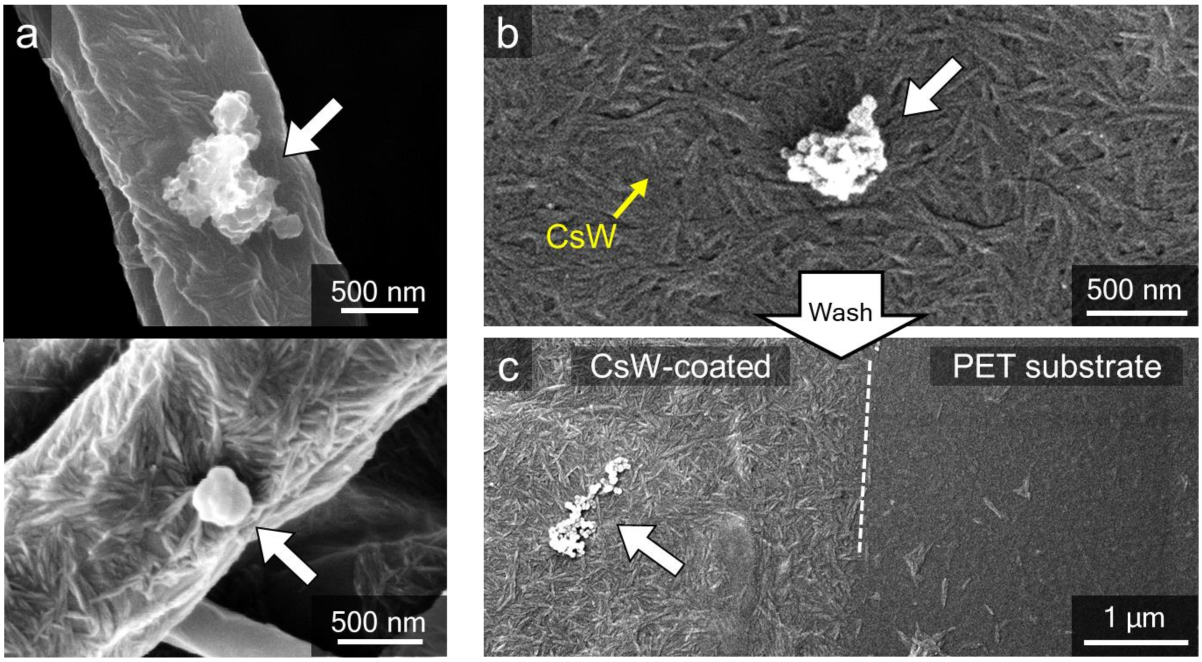

Figure S16. a) SEM images of a *bacteriophage Phi X174* cluster attached to CsW-coated PBS fibers. b, c) Well-attached viruses before and after washing. The white arrows indicate *bacteriophage Phi X174*.

Movie S1. Biodegradable and high-performance face mask filter.

Movie S2. PM blocking performance of Int-MN4.5.

Movie S3. Enzymatic filter degradation.

Reference

[S1] T. Kim, H. Jeon, J. Jegal, J. H. Kim, H. Yang, J. Park, D. X. Oh, S. Y. Hwang, *RSC Adv*. **2018**, *8*, 15389.
